# The Factor Structure and Rasch Analysis of the Fear of COVID-19 Scale (FCV-19S) Among Chinese Students

**DOI:** 10.3389/fpsyg.2021.678979

**Published:** 2021-09-22

**Authors:** Wei Chen, Yuxin Liang, Xingyu Yin, Xingrong Zhou, Rongfen Gao

**Affiliations:** ^1^School of Psychology, Guizhou Normal University, Guiyang, China; ^2^Center for Big Data Research in Psychology, Guizhou Normal University, Guiyang, China

**Keywords:** fear of COVID-19, factor structure, Chinese students, reliability, validity, differential item functioning, Rasch analysis

## Abstract

The Fear of COVID-19 Scale (FCV-19S) is a new one-dimensional scale used to measure fear of an individual about the COVID-19. Given the seriousness of the COVID-19 situation in China when our study was taking place, our aim was to translate and examine the applicability of the FCV-19S in Chinese students. The sample used for validation comprised 2,445 Chinese students. The psychometrical characteristics of the Chinese FCV-19S (FCV-19S-C) were tested using Rasch analysis. Principal component analysis (PCA) proved the unidimensional structure of the model. Both infit and outfit mean square (MNSQ) values (0.69–1.31) and point-measure correlations (0.82–0.86) indicated a good model fit. Person-item separation and reliability values indicated good reliability of the scale. The person-item map revealed an acceptable level of match between the persons and the items. Differential item functioning of the FCV-19S-C showed no differences with respect to age or gender. FCV-19S-C scores were significantly associated with anxiety, stress, depression, ego-resilience, and general health. The FCV-19S-C was proven to be effective in measuring fear of Chinese students about the COVID-19.

## Introduction

In December 2019, multiple cases of both respiratory and nonrespiratory infections were detected in China and were later identified as being COVID-19 (Rothan and Byrareddy, 2020). On January 30, 2020, the World Health Organization (WHO) declared a public health emergency of immediate concern (Wu and McGoogan, 2020). According to a WHO report published May 16, 2021, the number of new cases and deaths in the previous week had continued to decrease with just over 4.8 million new cases and just under 86,000 new deaths reported globally. Despite a declining trend over the previous 3 weeks at that point, the incidence of cases is nonetheless at some of the highest levels in various countries since the start of the pandemic (World Health Organization, 2021). At the time of publishing this article, it is still unclear when the pandemic will end. This uncertainty, along with the high infectivity rate of COVID-19 and a lack of transparency from clarifying, has resulted in millions of people worldwide feeling high levels of stress and seeing the harmful carry-on effects of stress on both individuals and societies. Some of the main symptoms of fear of COVID-19 include anxiety, depression, and traumatic stress (Zandifar and Badrfam, 2020).

Due to the high infectivity and fatality rates of COVID-19, many countries have implemented a number of measures to reduce the spread of the virus. Until vaccines are in widespread use, lockdowns and quarantine are understood to be the best way of controlling infection rates (Pakpour and Griffiths, 2020). However, this has led to severe interruptions of routines, separation of family and friends, shortages of daily necessities, wage losses, and school closures. While these emergency responses do limit exposure of people to the deadly virus, many who are in quarantine or lockdown experience boredom, loneliness, and insecurity on a large scale (Brooks et al., 2020). Thus, the potential negative impact of COVID-19 on public mental health must be taken seriously (Ornell et al., 2020). The mental health of students is one area of great concern. Students, in particular, have often received too much negative information regarding the virus and, as a result, shown a gradual emergence of anxiety, depression, and fear due to school closures and losses of well-developed social networks. For example, Cao et al. (2020) found that 2.7% of Chinese students had moderate anxiety, while 0.9% were suffering from severe anxiety during the pandemic. One recent survey found increased levels of stress or anxiety in 91% of U.S. college students (Active Minds, 2020). Colizzi et al. (2020) illustrated the negative effects of fear of COVID-19 using a case report of a patient who was not in fact infected with COVID-19, but exhibited COVID-19-like symptoms. Other recent studies have added further support to this claim, demonstrating that fear of COVID-19 may exacerbate levels of anxiety and depression (Ahorsu et al., 2020; Lee et al., 2020). The fear of COVID-19 can also cause delays to healthcare access and even lead to suicide (Goyal et al., 2020; Lazzerini et al., 2020). Another study revealed that fear of COVID-19 poses a risk for psychological resilience, in that a high level of resilience is the ability of an individual to protect their mental health and reduce the risk of infection (Seçer et al., 2020). Therefore, when adverse psychological troubles (e.g., fear) occur, certain interventions can effectively reduce and eliminate the negative effects of COVID-19 fear on mental health (Duan and Zhu, 2020; Xiang et al., 2020). As students are a demographic that is particularly vulnerable to these psychological problems, it is important to develop a method to accurately evaluate fear of a student about the COVID-19.

Fear of COVID-19 is a joyless mood state that is induced by perceived threat stimuli (Pakpour and Griffiths, 2020). The Fear of COVID-19 Scale (FCV-19S) was developed in an Iranian context to measure this fear and is a unidimensional scale composed of seven high-quality items to effectively measure the fear of an individual about COVID-19 (Ahorsu et al., 2020). It has subsequently been translated into numerous languages, and the one-factor structure, validity, and reliability have been confirmed in many national and regional contexts, Malaysia (*n* = 228; Pang et al., 2020), Turkey (*n* = 1,304, *n* = 668; Haktanir et al., 2020; Satici et al., 2020), the United States (*n* = 237; Perz et al., 2020), Bangladesh (*n* = 8,550; Sakib et al., 2020), Italy (*n* = 250; Soraci et al., 2020), and Arabia (*n* = 639; Alyami et al., 2020). In further development of this research, Bitan et al. (2020) proposed a two-factor structure in an Israeli sample. Meanwhile, a bifactor structure proved to be the best fit in the Ecuadorian sample (Moreta-Herrera et al., 2021). However, evidence from recent cross-nation measurement invariance studies showed that FCV-19S is one-dimensional in 11 countries (Lin et al., 2021), while another study found that the scale has a two-factor structure among seven Latin American countries (Caycho-Rodríguez et al., 2021). These differences in findings indicate that the factor structure of FCV-19S merits further research.

The FCV-19S has been used less in the Chinese context so far. Chang et al. (2020) examined the psychometric properties of three COVID-19-related scales [i.e., FCV-19S, Believing COVID-19 Information Scale (BCIS), and Preventive COVID-19 Infection Behaviors Scale (PCIBS)] among individuals with mental illness. A single-factor structure of the FCV-19S was confirmed by the confirmatory factor analysis (CFA). Meanwhile, Chi et al. (2021) compared three different factor structures (i.e., one-factor, two-factor, and bifactor) of the Chinese version of FCV-19S (FCV-19S-C) and found that a bifactor structure with a general fear factor and two orthogonal group factors with fear thoughts and physical responses showed the best fit. As the factor structure of FCV-19S-C is clearly controversial, the purpose of the current study was to further explore the factor structure of the scale in a Chinese student context. Pakpour et al. (2020) have recently supported the findings of Ransing et al. (2020) regarding the unstable factor structure of the FCV-19S and confirmed its unidimensional structure. Pakpour et al. (2020) were of the opinion that the two-factor structure as found by both Russian and Hebrew explorations of the measure was a consequence of their inappropriate use of principal component analysis (PCA) or exploratory factor analysis (EFA). Therefore, in order to resolve the factor structure arguments surrounding the FCV-19S-C, our study used a new method, namely, Rasch analysis, to verify the one-factor structure of the FCV-19S-C.

Rasch analysis can be used to address some limitations of classical test theory (CTT), such as sample dependency (Zile-Tamsen, 2017). In Rasch analysis, it is assumed that measurement errors are different in different individuals and not dependent on a particular sample. Furthermore, the Rasch model makes it easier to assess the validity of the latent structure of the scale by sharing a meterstick between the persons and the items (Rustøen et al., 2018). The Rasch model is commonly used in cross-cultural adaptation research, among which the differential item functioning (DIF) is an efficient method for testing cross-cultural validity (Watt et al., 2015). Therefore, we decided that Rasch analysis would be appropriate to explore the structure of the FCV-19S-C. Studies have found that females are more openly afraid of COVID-19 than males (Reznik et al., 2020). A study of Vietnamese medical students found that older students may have had lower levels of fear of COVID-19 during the pandemic (Nguyen et al., 2020). Taking into account the influences of gender and age, we decided to perform a DIF analysis to test whether the items in the FCV-19S-C were equivalent to those of the original Iranian survey (Petersen et al., 2003).

## Methods

### Participants

The target demographic for the current study was the general Chinese student population. The final sample included 2,445 Chinese students (mean age = 18.55; *SD* = 3.78; 49.8% males). The students ranged in age from the fourth grade in primary school to the third grade in doctorate studies (0.2% primary school, 14.7% junior high school, 38.4% senior high school, 44.5% undergraduates, 2.1% postgraduates, and 0.1% doctors). Of the total, 1,235 (50.5%) participants were 18 years of age and under, while 1,210 (49.5%) were older than 18 years.

### Procedure

Data for this study were collected in mid-May 2020. We selected one primary school, three middle schools, and one university in China to collect our subjects. All participants were students above grade four in primary school and could fully understand the meaning of the questions. After first contacting teachers at the school, the questionnaire was then shared with participants through the Chinese electronic platform “WJX.cn” and WeChat. Due to the infection prevention and control measures in place due to the pandemic, the school teachers distributed the questionnaire as a link in the class chat group, and explained the objectives, benefits, and risks of taking part, as well as the voluntary nature and confidentiality of the study. Students gave their informed consent by clicking the option that read, “I agree to participate.” After the participants had clicked the option, “I agree to participate,” the complete questions were presented; if the students did not choose that option, the survey was ended automatically. For children and teenagers under the age of 18, the questionnaire link was sent to the chat group of parents. Parents were able to read the informed consent on the front page of the questionnaire link. The study was approved by the committee of the School of Psychology of Guizhou Normal University (20200523).

### Adaptation of the FCV-19S

The FCV-19S scale was translated into Chinese following standardized international guidelines (Cha et al., 2007). First, two bilingual people with backgrounds in psychology translated the scale independently (Chinese versions V1 and V2). Two master's students majoring in English then translated the Chinese versions (V1 and V2) independently into English (V3 and V4). After this, two reviewers compared the original and back-translated versions of the measure. When they found differences between the two, they explained these to the translators in order for them to retranslate the items effectively. This process continued until the two reviewers agreed that the English versions are the same, which resulted in the final FCV-19S-C (V5).

### Measures

Demographic information. A background questionnaire asking for age, grade, and gender details was used to obtain demographic information about the participants.

Fear of COVID-19 Scale (FCV-19S). The measure was first developed by Ahorsu et al. (2020) and then used to reliably measure fear of COVID-19 among the various general population. The measure consists of seven items. Responses are measured on a 5-point scale, with options ranging from 1 (“strongly disagree”) to 5 (“strongly agree”). A higher total score indicates a greater fear of COVID-19.

Depression Anxiety Stress Scale (DASS-21). This was developed by Lovibond and Lovibond (1995) and translated into Chinese by Gong et al. (2010). The scale consists of three dimensions—depression, anxiety, and stress—each with seven items, and scored using on a 4-point scale, from 0 (“never”) to 3 (“always”). The higher the total score, the stronger the negative emotional experience of an individual. In this study, the Cronbach α of the scale was 0.972.

The Ego-Resilience Scale (ERS). This was developed by Block and Kremen (1996) and translated into Chinese by Yu and Zhang (2007). The scale comprises 14 items and is measured using a 4-point scale, with options ranging from 1 (“strongly disagree”) to 4 (“strongly agree”). The higher the total score of the scale, the stronger the mental ego-resilience of an individual. In this study, the Cronbach α was 0.944.

The General Health Questionnaire (GHQ-12). This was developed by Goldberg (1972) and uses 12 items to measure feelings of strain, depression, inability to cope, anxiety-based insomnia, and lack of confidence. The Chinese version has been used by Yang et al. (2003) among community settings in mainland China. Responses are measured using a 4-point scale, with options ranging from 0 (“not at all”) to 3 (“more than usual”). In this study, the Cronbach α was 0.853.

### Data Analysis Strategy

The mean, standard deviation, skewness, and kurtosis were each calculated. Byrne and Campbell (1999) have pointed out that a normal distribution is when skewness and kurtosis values are close to zero (i.e., between −1.5 and 1.5). Acceptable values of corrected item–total correlation are >0.4 (Pakpour et al., 2014).

The Rasch model assumes that the scale is unidimensional, and a PCA of the residuals can be used to test the unidimensionality (Williams et al., 2012). When an eigenvalue for the first contrast of residual is <2.0, or the variance proportion explained by the measure is ≥20%, the hypothesis can be supported.

The infit and outfit MNSQ for item and person parameters can be used to test the model fit, and values from 0.5 to 1.5 are acceptable (Linacre, 2006). Model items with inadequate fit should be removed from subsequent analysis.

The internal consistency reliability was measured using the Person Reliability Index (PRI) and the Item Reliability Index (IRI), with 0.8 representing the lowest acceptable value. To test whether the instrument was sensitive enough to distinguish between samples at different levels, the Person Separation Index (PSI) and Item Separation Index (ISI) were used to evaluate the measure, with a value >2 indicating a good model fit (Fox and Jones, 1998).

Person-item maps (Wright Maps) represent the relationship between a person and an item. Person ability measures, located on the left side of the map, represent a range of levels of fear of participants, from high to low. Item difficulty thresholds are located on the right of the map, showing a range of difficulty from high to low (Meyer, 2014).

With regard to gender and age influences, measurement invariance was represented by DIF based on the Rasch analysis (Wu et al., 2017). We checked each item to ascertain whether they performed differently in subgroups (e.g., females vs. males; students under 18 years of age vs. students older than 18). The DIF contrast (i.e., difference in difficulty of the item between the two groups) followed the following criteria: no DIF (<0.50 logits), minimal (0.50–1.0 logits), and notable (>1.0 logits; Khadka et al., 2014).

Finally, we also examined the correlations between the FCV-19S-C and external variables (i.e., anxiety, stress, depression, ego-resilience, and general health) by using Cohen's standards of weak (≤0.30), moderate (0.30–0.50), and strong (≥0.50; Cohen, 1988).

Except for the Rasch model analysis *via* WINPEPS 3.74.0, all analyses were performed using STATAMP 13.1.

## Results

### Descriptive Statistics

We could see that the kurtosis and skewness of the items did not conform to the normal distribution (see Table 1). Otherwise, the corrected item–total correlations of all items were far above the standard (0.824–0.900).

**Table 1:** Means, *SD*s, kurtosis, skewness, and corrected item–total correlation results of the FCV-19S-C (*n* = 2,445).

**Item**	**Mean**	***SD***	**Kurtosis**	**Skewness**	**Correlation**
1	2.968	1.314	1.784	−0.152	0.835
2	2.680	1.276	1.805	0.136	0.882
3	2.423	1.260	1.897	0.380	0.900
4	3.052	1.322	1.825	−0.265	0.824
5	2.907	1.291	1.767	−0.136	0.848
6	2.394	1.260	1.915	0.406	0.873
7	2.508	1.277	1.773	0.249	0.887

*SD, Standard deviation*.

### Unidimensional

The PCA of the residuals produced an eigenvalues of 14.8, and the proportion of variance explained by the measure was 67.9%. The value of the first contrast of residual was 1.9, lower than the acceptable maximum values of 2.0 (see Table 2).

**Table 2:** Variance of standardized residuals.

	**Eigenvalues**	**Observed (%)**	**Expected (%)**
**Goal setting**			
Total raw variance	21.8	100.0	100.0
Raw variance explained by measures	14.8	67.9	67.7
Raw variance explained by persons	9.6	43.9	43.8
Raw variance explained by items	5.2	24.0	23.9
Raw unexplained variance (total)	7.0	32.1	32.3
Raw variance unexplained in first contrast	1.9	8.7	27.1

### Model Fit of Items

Infit MNSQ and outfit MNSQ were acceptable by normal standards for all seven items (i.e., 0.69–1.31; see Table 3). In addition, the PT-measure correlation values ranged from 0.82 to 0.86. The positive and high correlations showed that the model fits expectations well.

**Table 3:** Model fit for each item.

**Item**	**Model SE**	**MNSQ**		**PT-measure**	
		**Infit**	**Outfit**	**Correlation**	**Expected**
1	0.03	1.25	1.23	0.82	0.85
2	0.03	0.87	0.89	0.86	0.84
3	0.03	0.73	0.69	0.86	0.83
4	0.03	1.31	1.24	0.82	0.85
5	0.03	1.12	1.07	0.83	0.85
6	0.03	0.87	0.88	0.84	0.83
7	0.03	0.82	0.81	0.85	0.83

### Reliability

The results demonstrated the promising separation reliability and index in the Rasch analysis (item separation reliability = 1.00, item separation index = 18.44, person separation reliability = 0.88, and person separation index = 2.77).

### Person-Item Map

**Figure 1:**
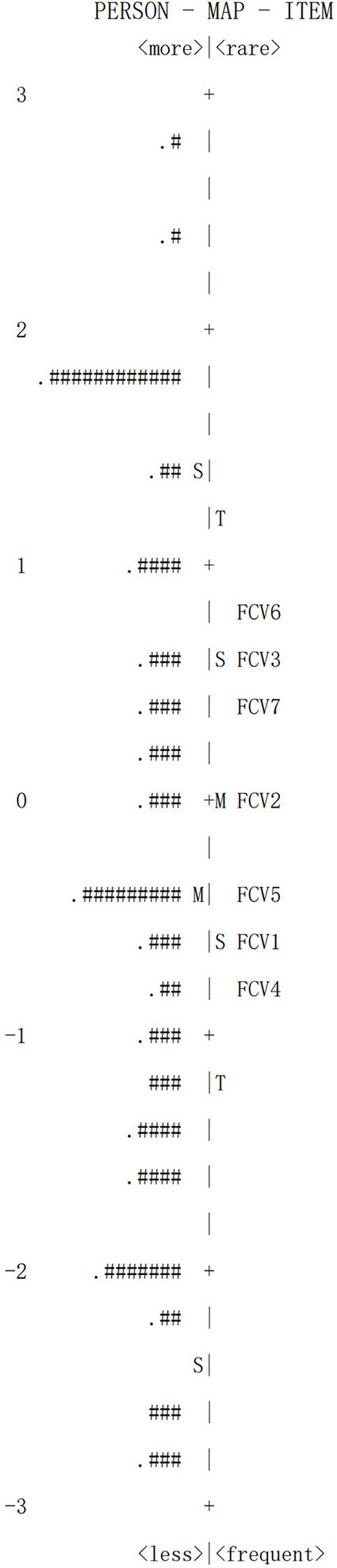
Person-item map for the FCV-19S.

Figure 1 shows the Person-Item Map (PIM), which indicates student ability and item difficulty in the logit scale ranging from −3 to 3. The top left indicates those who have high levels of fear, while the bottom left shows students with low levels of fear of COVID-19. Item difficulty is shown on the right side of the PIM. Students seldom answered “agree” to Fcv6 (“I cannot sleep because I'm worrying about getting coronavirus-19”), while the majority did “agree” to FCV5 (“When watching news and stories about coronavirus-19 on social media, I become nervous or anxious”).

### Differential Item Functioning

No DIF existed for the seven items, neither in age nor in gender groups. An independent sample *t*-test was used to determine the relationship between gender and fear score (see Table 4). The results show that males scored significantly lower in fear of COVID-19 than females (mean_female_ = 19.36 [*SD* = 8.16] vs. mean_male_ = 18.50 [*SD* = 7.33]; *t* = 2.7463, *p* < 0.05). In contrast, age did not correlate significantly with FCV-19S-C total scores (*r* = 0.001, *p* = 0.261).

**Table 4:** Differential item functioning (DIF) for gender and age.

**Item**	**DIF contrast across gender**	**DIF contrastacross age**
1	0.20	0.10
2	−0.23	0
3	−0.11	0.15
4	0.33	0.00
5	0.13	−0.39
6	−0.21	0.08
7	−0.09	0

*DIF contrast across gender, Differential Item Functioning for females and males; DIF contrast across age groups, Differential Item Functioning for younger (i.e., ≤ 18 years) and older (i.e., >18 years) students*.

### Criterion Validity

Based on findings from existing research, we selected anxiety, stress, depression, ego-resilience, and general health as external criteria. The average statistically significant correlation values of the FCV-19S-C with the DASS-21, ERS, and GHQ-12 were |*r*| = 0.487, 0.322, and 0.499, respectively. More specifically, the average statistically significant correlation values of the FCV-19S-C with the three factors of the DASS-21 (i.e., depression, anxiety, and stress) were |*r*| = 0.467, 0.482, and 0.469, respectively. As expected, the FCV-19S-C had a moderate positive correlation with anxiety, stress, depression, and general health. Conversely, the FCV-19S-C had a moderate negative correlation with ego-resilience. These results indicate that the FCV-19S-C has good convergent and discriminant validity.

## Discussion

The purpose of this study was to examine the applicability of FCV-19S-C in Chinese students by using Rasch analysis. Our results show that FCV-19S-C has a stable one-dimensional structure and strong internal consistency. The moderate correlations with the DASS21, GHQ-12, and ERS also revealed good criterion validity. Invariance of the scale items was also confirmed between different age and gender groups.

The results of the PCA of the residuals indicate that the FCV-19S has good unidimensionality for Chinese students and is consistent with the single-factor structure of the original scale (Ahorsu et al., 2020). The original FCV-19S was developed based on Protective Motivation Theory (Rogers, 1975), but the two-factor and bifactor structures proposed by other studies are not supported theoretically. In view of the clarification of factor structure by the original author of the scale, our study also further proved the stability of one-factor structure (Pakpour et al., 2020).

The infit, outfit MNSQ, and PT-measure correlation values show that all items of FCV-19S-C are highly compatible with the Rasch model. It was documented that the infit and outfit MNSQ values can assist in demonstrating the unidimensionality of the scale (Brentari and Golia, 2007). Therefore, these values also support the results of PCA in that the FCV-19S-C is a good unidimensional measurement.

Rasch analysis evaluates the reliability of both the person measures and the items of the instrument. This is a great advance over what has been done in the past with the computations of Cronbach's alpha or KR-20 and avoids the flaw of using raw data for reliability evaluation (Linacre, 1997). The PRI and IRI were 0.88 and 1.00, respectively, showing high reliability values for persons and items and exceeding the minimum standard (0.70). Using PSI and ISI, we are able to assess (1) how well a set of items is able to differentiate different participants and (2) how well the set of items is able to be differentiated by the group of participants. The PSI and ISI values of this study were both higher than the recommended standards (1.5), indicating that the FCV-19S-C is sensitive enough to distinguish between high and low participants. Furthermore, the sample size in the current study is large enough to confirm the item difficulty hierarchy of the instrument (Fox and Jones, 1998).

The value of the PIM lies in how it assesses the ability of participants to cope with item difficulty (Englehard, 2013). The item distribution of the scale was basically consistent with the distribution of the latent trait level of the participant in PIM. This indicates that the FCV-19S-C is suitable for use in measuring student sample and that the items in the scale can effectively distinguish the level of fear of an individual.

The results of the current study show that the FCV-19S-C is an instrument without DIF for gender and age, which is consistent with previous studies assessing the FCV-19S (Ahorsu et al., 2020; Pang et al., 2020; Sakib et al., 2020). Hence, our findings support the stability of the FCV-19S-C in measuring psychological fear of Chinese students about COVID-19, without being influenced by gender or age. The current study found that males reported a lower level of fear of COVID-19 than females. Fear can be an emotional response to an external factor. It is not a new phenomenon to see gender differences in reactions to an external threat; for example, one previous study showed that women are generally more afraid of pollution and aversion to sensitivity than men (Olatunji et al., 2005).

The results of the current study are similar to those from the previous Malay validation studies (Pang et al., 2020), which have used depression, anxiety, and stress scales to test for concurrent and criterion validity. Overall, the positive correlations between depression, anxiety, stress, and the FCV-19S score have been proven in previous related studies. In other words, fear of COVID-19 appears to have a direct effect on levels of depression, stress, and anxiety (Yildirim et al., 2020). Resilience may help individuals achieve better mental health by buffering the effects of fear on mental health problems in adversity (Yildirim, 2019). The current study also reveals a significant negative relationship between fear and resilience. All results are promising in alleviating the negative impact of fear of Chinese students about COVID-19 and providing evidence of psychological protection.

### Limitations and Future Directions

There are some limitations in study. First of all, this study only used a self-report scale to evaluate degree of fear of participants. This angle is subjective and is also vulnerable to other factors, such as social expectations and method bias. Other research methods should also be used to explore levels of fear in future studies (e.g., in-depth interviews). Second, the design of the current study was cross-sectional and did not examine the stability of the FCV-19S-C over time. Longitudinal studies should also be done to assess the relationship between fear of COVID-19 and other influencing variables over time.

## Conclusion

This study used Rasch analysis to demonstrate that the FCV-19S-C has good psychometric performance and can effectively measure the psychological fear of COVID-19 in the Chinese student population.

## Data Availability Statement

The original contributions presented in the study are included in the article/Supplementary Material, further inquiries can be directed to the corresponding author/s.

## Ethics Statement

The studies involving human participants were reviewed and approved by Ethics Committee of Guizhou Normal University of Psychology (20200523). Written informed consent to participate in this study was provided by the participants' legal guardian/next of kin.

## Author Contributions

WC concepted the article and provided framework of the manuscript. YL analyzed the data and drafted the manuscript. XY and XZ collected the data. RG offered suggestions and guidance for revising the data analysis of this manuscript. The final version was approved by WC. All authors contributed to the article and approved the submitted version.

## Funding

This research was funded by the Natural Science Research Funding Project of Department of Education Guizhou Province (Grant No. Qian Ke He KY Zi [2021]299), 贵州教育改革发展研究重大项目(ZD202009), 贵州省教育厅高等学校人文社会科学研究项目(2022ZD006), and 贵阳市白云区科技计划项目(白科合同[2016]63号).

## Conflict of Interest

The authors declare that the research was conducted in the absence of any commercial or financial relationships that could be construed as a potential conflict of interest.

## Publisher's Note

All claims expressed in this article are solely those of the authors and do not necessarily represent those of their affiliated organizations, or those of the publisher, the editors and the reviewers. Any product that may be evaluated in this article, or claim that may be made by its manufacturer, is not guaranteed or endorsed by the publisher.
